# ASSESSMENT OF MECHANICAL AND MANUAL SUTURE IN THE SURGICAL TREATMENT OF THE PHARYNGOESOPHAGEAL DIVERTICULUM

**DOI:** 10.1590/S0102-6720201500040005

**Published:** 2015

**Authors:** José Luis Braga de AQUINO, José Francisco Salles CHAGAS, Marcelo Manzano SAID, Maria Beatriz Nogueira PASCOAL, Luis Antonio BRANDI-FILHO, Douglas Alexandre Rizzanti PEREIRA, Fernanda FRUET

**Affiliations:** Thoracic, Head and Neck Surgery Department, Hospital and Maternity Celso Pierro, Medical School, Pontifical Catholic University of Campinas, Campinas, SP, Brazil

**Keywords:** Zenker's diverticulum, Diverticulectomy, Mechanical suture

## Abstract

**Background:**

The occurrence of the pharyngoesophageal, or Zenker diverticulum is not frequent in the national scenario, and the technique of the diverticulectomy with cricomyotomy in medium and great dimension diverticula is still the most indicated. Because the resection of the diverticulum requires the suture of the pharynx, dehiscence can occur, thereafter delaying swallowing. Hence, the idea is to accomplish this surgical procedure, comparing the manual and mechanical suture, in order to evaluate the real benefit of the mechanical technique.

**Aim:**

To evaluate the results of the pharyngoesophageal diverticulectomy with cricomyotomy using manual and mechanical suture with regard to local and systemic complications.

**Method:**

Fifty-seven patients with pharyngoesophageal diverticula diagnosed through high digestive endoscopy and pharyngeal esophagogram were studied. The applied surgical technique was diverticulectomy with myotomy of the cricopharyngeal muscle, done in 24 patients (42.2%) the mechanical suture (group A) with the mechanical linear suture device and in 33 (57.8%) a manual closure of the pharynx (group B).

**Results:**

In the postoperative period, one patient of group A (4.1%) presented fistula caused by dehiscence of the pharyngeal suture, and three of group B (15.1%) presented the same complication, with a good outcome using a conservative treatment. In the same group, three patients (9.0%) presented stenosis of the suture of the pharynx, with good outcome and with endoscopic dilatations, and no patient from group A presented such complication. Lung infection was present in five patients, being two (8.3%) of group A and three (9.0%) on B, having good outcomes after specific treatment. In the late review, done with 43 patients (94.4%) of group A and 22 (88.0%) on B, the patients declared to be pleased with the surgical procedure, because they were able to regain normal swallowing.

**Conclusion:**

The diverticulectomy with myotomy and pharyngeal closure using mechanical suture was proven appropriate, for having restored regular swallowing in most of the patients, and the mechanical closure of the pharynx proved to be more effective in comparison to the manual one, because it provided a lower index of local post-surgical complications.

## Abstract (pt)

**Racional:**

A ocorrência do divertículo faringoesofágico, ou de Zenker, é pouco frequente no cenário nacional, sendo que a técnica da diverticulectomia com cricomiotomia em divertículos de média e grandes dimensões ainda é a mais indicada. Devido à ressecção do divertículo necessitar de sutura da faringe ocorre possibilidade de deiscência, o que retarda o retorno da deglutição. Daí a ideia de realizar este procedimento cirúrgico, comparando a sutura manual com a mecânica, para avaliar o real benefício da técnica mecânica.

**Objetivo:**

Avaliar os resultados da diverticulectomia faringoesofágica com cricomiotomia utilizando à sutura manual e mecânica em relação às complicações locais e sistêmicas.

**Métodos:**

Foram estudados 57 pacientes com divertículos faringoesofágicos diagnosticados através da endoscopia digestiva alta e faringoesofagograma. A técnica cirúrgica empreendida foi a diverticulectomia com miotomia do músculo cricofaríngeo, sendo a sutura mecânica realizada em 24 pacientes (42,2%, grupo A) com o aparelho linear e em 33 (57,8%, grupo B) a manual para o fechamento da faringe.

**Resultados:**

Na avaliação do pós-operatório precoce, um paciente do grupo A (4,1%) apresentou fístula consequente à deiscência da sutura da faringe e três do grupo B (15,1%) ambos com boa evolução com tratamento conservador. Neste mesmo grupo, três pacientes (9,0%) apresentaram estenose da sutura da faringe, com boa evolução com dilatações endoscópicas sendo que nenhum do grupo A apresentou esta complicação. A infecção pulmonar esteve presente em cinco pacientes, dois (8,3%) do grupo A e três (9,0%) do grupo B, com boa evolução com tratamento específico. Na avaliação tardia, realizada em 43 pacientes, 17(94,4%) do grupo A e 22 (88,0%) do grupo B, os pacientes referiram estarem satisfeitos com o procedimento cirúrgico, pois conseguiram resgatar a deglutição normal.

**Conclusões:**

A diverticulectomia com a miotomia do cricofaríngeo demonstrou ser procedimento cirúrgico adequado por restaurar deglutição adequada e o fechamento da faringe com sutura mecânica mostrou-se técnica mais eficaz em relação à sutura manual, com menor índice de complicações pós-operatórias locais

## INTRODUCTION

Although the dates are somewhat divergent among the published studies, the pharyngoesophageal diverticulum was first described by Abraham Ludlow in 1764^17^. However, it was only in 1877 that this disease was minutely studied by the German pathologist Albert Zenker^27^, who possessed considerable data and through his studies was able to correlate the clinical and anatomopathological aspects of this disease, besides giving the name of it. Nevertheless, the first successful resection of Zenker's diverticulum was performed by Whealer^1^ and it only occurred in 1886. 

Zenker's diverticulum basically consists of a dilated saccular deformation, located in the lower posterior wall of the pharyngeal mucosa, above the upper esophageal sphincter over a region located between the obliquely striated muscular fibers of the lower constrictor muscle of the pharynx and the transverse fibers of the cricopharyngeal muscle, also known as Killian's triangle. This region is more predisposed to herniation of the mucosa due to the high intraluminal pressure over this vulnerable area, in which the muscular fibers are more scarce, thus exposing the hypopharyngeal mucosa^1^^,^ 27.

The pharyngoesophageal diverticulum is not a very frequent disease among the population, being responsible for 1 to 3% of the complaints of dysphagia and 4% of patients with esophagus disease^1^^,^ 21. Its prevalence is more significant between the ages 60 to 80 years old, with its peak of incidence about the age of 70, being rare before the age of 40^1^^,^ 21. This is due to the loss of muscle tone and the decrease of resistance of the rear wall that returns physiologically with aging. The disease is more predominant among males in the proportion 3:1^5^ . Its occurrence is more common in countries in the North of Europe, being extremely rare in the countries in the far eastern countries. There are few studies pointing at the exact occurrence of Zenker's diverticulum in South American countries, including Brazil, but is known that it is not a common disease among the population^1^^,^ 2 ^,^ 21.

Patients with this disease present dysphagia and regurgitation as main symptoms, and they may also present halitosis and weight loss as secondary symptoms, which affect their life quality significantly^21^^,^ 23 ^,^ 25. 

The diagnosis can be done through a minute clinical investigation, complemented by doing barium contrast radiographic examinations of the pharynx and the esophagus, by the direct visualization of the esophagus through high digestive endoscopy, and if it is necessary, manometry can also be done^1^. 

The treatment is fundamentally surgical, with diverticulectomy or diverticulopexy, followed by cricopharyngeal myotomy, although in the past years, some authors support the endoscopic treatment^1^^,^ 16 ^,^ 19 ^,^ 22. Although diverticulectomy is a well standardized procedure, it is not free from complications, being the cervical fistula caused by dehiscence of the pharyngeal suture the most common type^15^^,^ 21. Although this complication is usually solved with conservative treatment, it compromises the life quality of patients, for delaying swallowing and thus interfering with the patient's nutrition. 

With the advent of mechanic suture demonstrating to be safe and accurate, it started to be used in many segments of the gastrointestinal tract for benign or malignant diseases^4^^,^ 5. This type of suture demonstrated the possibility of minimizing the complications referring to anastomosis, because it presents two plans, inverting and reducing ischemia and tissue necrosis^4^. 

 Little national emphasis has been given on the use of mechanical suture in the closing of the pharynx after diverticulum resection, except for a recent study done by Aquino et al.^6^, which demonstrated good results with this kind of procedure. However, there was no comparison of this type of suture with the manual type to evaluate whether the mechanical technique would be more advantageous. 

Therefore, the objective of this study was to evaluate the results of the surgical treatment of the pharyngoesophageal diverticulum, through diverticulectomy with the cricopharyngeal myotomy, comparing the linear mechanical suture with the manual suture in the closing of the pharynx regarding their systemic and local complications. 

## METHOD

### Patients 

From January of 1994 to December of 2013, 57 patients having the diagnosis of pharyngoesophageal diverticulum in the Thoracic, Head and Neck Surgery Department of the Hospital and Maternity Celso Pierro from the Pontifical Catholic University of Campinas were analyzed, and were eligible for a proposed surgery. Forty-two (73,6%) were male and 15 (26,4%) female, with age ranging from 56 to 89 years old (67,5).

### Preoperative evaluation

The diagnosis was done through clinical, radiological and endoscopic evaluations. In the clinical evaluation, the most relevant observed symptoms were dysphagia for solids from four to seven years intermittently in all patients; 39 patients (68.8%) presented weight loss; periodical regurgitation was present in 36 patients (63.1%) and being associated with cough in 23 of them (40.3%); 36 patients were smokers of one pack of cigarettes per day with variable time ranging from 35 to 54 years. Twenty-seven (47.3%) reported to drink one serving of alcoholic distillate per day with variable time ranging from 25 to 47 years. 

The pharyngeal esophagogram test was performed in every patient, confirming the presence of the pharyngoesophageal diverticulum.

The high digestive endoscopy showed in all patients the diverticula with medium and great dimensions, within 3 to 9 cm. This exam also evaluated that there were no diseases associated with the diverticulum in any of the patients. 

In all patients, the clinical and nutritional evaluations demonstrated that they were able to be submitted to the proposed surgical procedure. 

### Surgical technique

All patients were submitted to diverticulectomy and cricopharyngeal myotomy according to the following surgical tactics: 1) left supraclavicular neck incision and detachment of skin flap; 2) exposure of the left sternocleidomastoid muscle and dissection of its medial portion with exposure of the pharynx and cervical esophagus; 3) identification of the diverticulum and its dissection and the dissection of adjacent structures as far as the exposure of its floor together with the pharynx wall; 4) section of the diverticulum and closure of the pharynx; 5) cricopharyngeal myotomy until de proximal cervical esophagus with 3 cm of extension; 6) placement of nasogastric tube for immediate postoperative feeding; 7) placement of drain in the cervical region and closure of incisions. 

For the confection of the pharynx suture, the patients were distributed between two groups according to the technique applied: group A - mechanical suture with the linear device TA 45mm was done in 24 patients (42,2%); group B - manual suture with Vicryl3-0 was done in 33 patients (57,8%) being the first as continuous suture, involving all layers of the pharynx and the second, interrupted suture involving the muscular.

### Postoperative evaluation

The postoperative evaluation considered the observation of the following variables: 1) systemic complications: notably of cardiovascular, respiratory or infectious origins investigated daily by clinical improvement of the patients and by the results of laboratory and imaging exams that were requested; 2) local complications: stenosis and principally dehiscence of the pharyngeal suture, with fistula; 3) life quality: in this item, the postoperative day was considered, in which the patients started with normal swallowing and in case of dysphagia, its level was evaluated if it were mild (solid food), moderate (pasty food) and intense (liquids). 

Diagnosis can be reached through clinical observation, by the visualization of the output of salivary secretion around the cervical region until the 5^th^ postoperative day. In the absence of clinical evidence of fistula in the anastomosis, a pharyngeal esophagogram was performed in the 5^th^ postoperative day, to observe if there was contrast extravasation. In case of a negative result, liquid oral diet was permitted, evolving to pasty and solid diet, according to patient's acceptance. 

Regarding stenosis of the pharynx suture, the diagnosis was clinical, directed by symptoms of dysphagia from the 30^th^ postoperative day and the decrease of the pharyngeal lumen, proven by contrasted radiography and high digestive endoscopy.

## RESULTS

### Early assessment 

In the 30^th^ postoperative day, six patients (10.5%) presented fistula caused by dehiscence of the pharyngeal suture translated by the output of digestive secretion by the cervical drain from the 3^rd^ to 5^th^ postoperative days. Among the patients that presented this complication, one (4.1%) belonged to the mechanical suture group and five (15.1%) to the manual one. As there was no systemic repercussion consequent to this complication, conservative treatment was applied in all patients, with nutritional support by enteral diet and local bandage being done daily and achieving scarring of the fistulas between the 14^th^ to 23^rd^postoperative days. In these days, the contrasted pharyngeal esophagogram was done, and it did not show evidence of contrast extravasation in the pharynx suture in any of the patients. Thus, the oral diet was introduced initially with liquids, with progressive substitution to pasty and solid diets, being well accepted by patients. In the other 51 patients, 23 of group A and 28 of group B, in which there was no clinical evidence of dehiscence of the pharyngeal suture for the lack of output of digestive secretion by the cervical drain until the 5^th^ postoperative day, the contrasted exam was also done, and it did not demonstrate fistula in the pharynx. The oral diet was then introduced, progressing to liquids and then solids, being well accepted by patients. 

Five patients (8.7%) presented pulmonary infection, two (8.3%) belonging to group A and three (9%) to group B, and all of them presented good improvement with specific clinical treatment. All of the patients who had this complication suffered from chronic obstructive pulmonary disease and were long-term smokers. 

Dysphonia was present in four (7.0%) patients, two from each group. In three it was temporary, being reversed within 23 postoperative days and remaining in one patient, requiring rehabilitation from the Speech Therapy Department with small recovery; this patient belonged to group B. 

Although there was no dehiscence of the pharyngeal suture in three patients (5.2%), one (4.1%) belonging to group A and two (6,6%) to group B, they developed wound infection, being reversed by local drainage of the surgical incision. 

No patient died. 

### Mid and long term assessment

It was done in 43 (75.4%) patients, 18 belonging to group A and 25 to B, with time ranging from two months to 16 postoperative years (average of 5.4 years). During this assessment, three patients (9.0%), all from group B, presented moderate dysphagia between 65 to 80 postoperative days. The pharyngeal esophagogram test and high digestive endoscopy demonstrated stenosis of the suture of the pharynx. Four to seven sessions of endoscopic dilatation were done with good outcome. Intermittent regurgitation was also present in three patients (6.9%) two belonging to group A (11.1%) and one to B (4.0%). Seventeen patients (94.4%) from group A as well as 22 (88.0%) from group B reported to be satisfied with the surgical procedure, because they presented normal swallowing, obtaining significant better life quality.

## DISCUSSION

The occurrence of the pharyngoesophageal diverticulum is not frequent in our scenario; therefore, few are the departments that have enough patients to provide them with a satisfactory handling and treatment. 

The treatment of this disease is fundamentally surgical, being based on its ethiopathogenesis in such way that most authors have been practicing diverticulectomy followed by myotomy of the cricopharyngeal muscle^1^^,^ 6 ^,^ 9 ^,^ 11 ^,^ 26. Other authors have been practicing diverticulopexy, associated with cricopharyngeal myotomy, demonstrating similar results when compared with diverticulectomy and myotomy^14^^,^ 15 ^,^ 18. More recently, some authors have been practicing diverticulopexy in older patients with severe clinical comorbidity and with small diverticula, usually smaller than 3 cm^12^^,^ 14 ^,^ 15.

This is the reason that here was indicated the resection of the diverticulum associated with cricopharyngeal myotomy to these patients, as endoscopic evaluation showed that all of the diverticula were of 3 cm or larger; although the average age was of 67.5 years old, they did not present severe clinical comorbidity.

Another indication for the diverticulum resection was to prevent malignant transformation and potential in situ carcinoma^3^. 

The endoscopic treatment of pharyngoesophageal diverticulum also has many supporters and great experience is required to do it, which consists of dividing the septum between the diverticulum and the esophagus under endoscopic control^8^^,^ 24. Van Overbbek^24 ^reports endoscopic treatment results in 545 patients during 30 years, obtaining satisfactory improvement of dysphagia in 91% of them, with very low rates of complications. 

Ishioka et al.^13 ^reported their experience with a fiber endoscope to perform the septum section in 42 patients with Zenker's diverticulum, obtaining positive results, with 7.1% dysphagia recurrence.

As for diverticulectomy complications, the cervical fistula caused by dehiscence of the pharyngeal suture has been reported with variable incidence of 5-35.0%^1^^,^ 10 ^,^ 13 ^,^ 15 ^,^ 20 ^,^ 21 ^,^ 25. Although this complication is usually solved with conservative treatment, with drainage of the surgical incision with daily bandages and nutritional support by enteral catheter, it compromises the life quality of the patient for delaying oral swallowing. 

Thus, the advantage of mechanical suture, being inverted and double favors a better coaptation of the suture borders and minimizes this complication. This was well demonstrated in this study, because only 4.1% of group A presented dehiscence of the pharyngeal suture, whereas 15.1% of B had it. Although they did present improvement with conservative treatment, it took the latter patients more time to regain swallowing. Another advantage of mechanical suture is that no patient from this group progressed to suture stenosis, whereas three patients (9.0%) of the manual had it. Although it did not progress to any other expressive morbidity, it compromised swallowing in these patients, requiring the need for endoscopic dilatation. 

Bonavina et al.^7 ^also emphasized the advantages of mechanical suture in the closure of the pharynx after diverticulum resection, because, none of the 116 patients who underwent this procedure presented cervical fistula. 

Because the disease usually affects elderly patients with potential cardiopulmonary comorbidity, this condition predisposes postoperative systemic complications, and this fact was present in 8.7% of the patients in this series and was similar in others^1^^,^ 15 ^,^ 20 ^,^ 21 ^,^ 23 ^,^ 26. Smoking is another relevant factor, because all of the patients who presented this complication were smokers for years. 

In the mid and long term assessments, many authors have been demonstrating that diverticulectomy with cricopharyngeal myotomy promotes the disappearance of dysphagia's symptoms in most patients 1 ^,^ 10 ^,^ 12 ^,^ 14 ^,^ 16 ^,^ 20 ^,^ 21 ^,^ 23 ^,^ 26. This has also been well demonstrated in this study, because most of the patients from both groups regained proper swallowing. 

Andreollo et al.^1^ evaluating 38 patients who underwent surgical treatment of Zenker's diverticulum with average follow-up of 14 months, emphasized the advantages of the diverticulectomy with cricopharyngeal myotomy, because the group that underwent this procedure obtained excellent results of 84.6%, compared with 66.6% of the group that did the diverticulopexy and myotomy.

## CONCLUSION

Diverticulectomy with cricopharyngeal myotomy is a surgical procedure with great validity for providing most patients with proper swallowing; the mechanical suture seems to offer advantages if compared with the manual one, for having demonstrated lower rates of local complications, notably dehiscence and stenosis of the pharyngeal suture.
